# Simulation device for shoulder reductions: overview of prototyping, testing, and design instructions

**DOI:** 10.1186/s41077-023-00246-3

**Published:** 2023-03-09

**Authors:** Sorab Taneja, Will Tenpas, Mehul Jain, Peter Alfonsi, Abhinav Ratagiri, Ann Saterbak, Jason Theiling

**Affiliations:** 1 Duke Engineering Design Pod, 308 Research Dr, Durham, NC 27710 USA; 2 Department of Biomedical Engineering, Duke University, 101 Science Dr, Box 90281, Durham, NC 27708 USA; 3 Department of Surgery, Box 3096, Durham, NC 27710 USA

**Keywords:** Shoulder reduction, Simulation device, Traction-countertraction, External rotation maneuver

## Abstract

**Background:**

Shoulder dislocations are common occurrences, yet there are few simulation devices to train medical personnel on how to reduce these dislocations. Reductions require a familiarity with the shoulder and a nuanced motion against strong muscle tension. The goal of this work is to describe the design of an easily replicated, low-cost simulator for training shoulder reductions.

**Materials and methods:**

An iterative, stepwise engineering design process was used to design and implement ReducTrain. A needs analysis with clinical experts led to the selection of the traction-countertraction and external rotation methods as educationally relevant techniques to include. A set of design requirements and acceptance criteria was established that considered durability, assembly time, and cost. An iterative prototyping development process was used to meet the acceptance criteria. Testing protocols for each design requirement are also presented. Step-by-step instructions are provided to allow the replication of ReducTrain from easily sourced materials, including plywood, resistance bands, dowels, and various fasteners, as well as a 3D-printed shoulder model, whose printable file is included at a link in the Additional file 1: Appendix.

**Results:**

A description of the final model is given. The total cost for all materials for one ReducTrain model is under US $200, and it takes about 3 h and 20 min to assemble. Based on repetitive testing, the device should not see any noticeable changes in durability after 1000 uses but may exhibit some changes in resistance band strength after 2000 uses.

**Discussion:**

The ReducTrain device fills a gap in emergency medicine and orthopedic simulation. Its wide variety of uses points to its utility in several instructional formats. With the rise of makerspaces and public workshops, the construction of the device can be easily completed. While the device has some limitations, its robust design allows for simple upkeep and a customizable training experience.

**Conclusion:**

A simplified anatomical design allows for the ReducTrain model to serve as a viable training device for shoulder reductions.

**Supplementary Information:**

The online version contains supplementary material available at 10.1186/s41077-023-00246-3.

## Introduction

Shoulder dislocations in the USA impact an estimated 23.9 per 100,000 people [1]. Shoulder dislocations make up approximately 50% of all major joint dislocations, with anterior dislocations being most prevalent [2]. Up to 90% of these dislocations are managed in the emergency setting [2]. As such, it is imperative that emergency physicians are able to both diagnose and effectively treat shoulder dislocations.

If surgery is not required, a physician reduces the glenohumeral joint by maneuvering the humeral head back into the glenoid cavity [3]. There are many closed reduction methods, including the external rotation maneuver, the traction-countertraction method, scapular manipulation, the Bokor-Billmann technique, and the Cunningham technique [3, 4]. For all shoulder reduction techniques, manipulating the glenohumeral joint requires both considerable force as well as nuanced navigation [3].

Like many medical procedures, shoulder reduction methods are taught in classrooms but are most effectively learned through deliberate practice [5]. Typically, learning a new procedure involves three steps for the trainee: (1) learning the theory, (2) observing in a clinical setting, and then (3) treating a patient. While practice with a physical model or virtual simulation before direct patient interaction has become customary for some procedures, it is still absent in training for shoulder reduction [6, 7].

Over the past several decades, a handful of models and simulators have been developed in an effort to improve educational practices regarding shoulder reductions. American-based company Sawbones developed and previously sold a shoulder dislocation model that featured a replicated humeral head, glenoid fossa, and acromion [8, 9]. When placed on the floor or table, dislocations and subsequent reductions could be mimicked by moving the humeral head in and out of position either anteriorly and posteriorly [8, 9]. Tension could also be adjusted by turning a knob attached to the model [9]. While the model provided an accurate feel based upon the materials used to make the model, its prior retail price was high, and it is no longer for sale.

Other efforts were put forth by various groups, such as those at Loma Linda University Health and the Czech Technical University [8, 10, 11]. The former developed a shoulder reduction trainer that both looks like a real-life shoulder and can be used to practice a variety of reduction techniques [10]. Using a modification of a skeletal model of the right upper extremity, the team created an anatomical model that is assisted with rubber bands and drilled holes [10]. The model requires another person to wear the device for it to be used [10]. The group at Czech Technical University developed a computational model that utilized quantitative data and suggestions for parts that can be used to adequately replicate a shoulder reduction [8]. Despite its anatomical and physiological accuracy, it is merely a simulation and has not been used in practice [8]. Other models, such as an ultrasound-guided injection training for anterior shoulder reductions, have also been developed [11].

Given the landscape of shoulder reduction models, there is a need for a low-cost simulation device that supports the instruction and practice of shoulder reduction methods that can be easily built by practitioners and trainers. Thus, the aim of this work is to build a do-it-yourself (DIY) shoulder reduction model that supports the traction-countertraction and external rotation techniques. The new trainer should share many of the previously mentioned trainers’ strengths in that it adequately replicates the feel of various shoulder reduction techniques, yet also addresses weaknesses of the aforementioned trainers, including limitations in types of reduction techniques, cost, and possibility to be replicated autonomously with limited tools and time. In this paper, the team demonstrates how the novel ReducTrain device was created, shining light upon the iterative design process used to create the device. Additionally, the team justifies design choices and features and demonstrates how the model met testing goals. Finally, the paper explains ReducTrain’s uses and limitations in a training setting.

## Design and development process

The team developed the ReducTrain simulator through the iterative engineering design processes [12, 13]. The primary function of the model is to simulate the traction-countertraction and external rotation techniques, although other techniques may be used. Another guiding objective was that ReducTrain could support classroom instruction as a simplified visual representation of the shoulder. The ReducTrain is not anatomically identical to a shoulder or arm; rather, the model is designed to simulate the “feel” of the reduction process and mimic the varied pathways medical personnel maneuver the shoulder. This trade-off in anatomical accuracy allows the design to meet the other important objectives of being inexpensive, easy to assemble, and durable.

### Needs assessment

The team started with a needs assessment that included interviews with emergency medicine educators at Duke University Hospital about the current landscape for shoulder reductions and related training models. Based on multiple conversations, and considerations of the most pressing educational needs, the design team selected two reduction techniques: traction-countertraction and external rotation. Anterior and posterior dislocation regions were included on the traction-countertraction assembly, even though a nonsurgical reduction of a posterior dislocation is not common. The team then learned the motions from several emergency medicine practitioners who regularly treat shoulder dislocations. These practitioners showed approximate movements on themselves and team members. While going through these movements, the practitioners verbally described what they would be feeling in a real procedure. Research was also conducted on the current landscape of training techniques to understand gaps. The team used this information to formulate appropriate design requirements and realistic acceptance criteria.

### Design requirements and acceptance criteria

Next, the team established three design requirements, which are target features or characteristics that guided the design process. Table 1 lists the requirements of durability, ease of assembly, and cost, with their respective acceptance criteria. Durability was the ability of the model to withstand training conditions without needing any upkeep or adjustments. The goal was to make a durable device that did not need to have components replaced or be repaired before 1000 uses. Ease of assembly was focused around the device being easy to build for somebody with access to basic hardware supplies and a makerspace or workshop. Cost was a critical factor to ensure the device filled the existing gap of affordable, free-standing simulators [8]. Simulation face validity was also considered and defined as how realistic the simulation was in relation to the feel of a real reduction. The team did not evaluate this because of the complexity required to study this and leaves it to future work as noted later.

**Table 1:** Design requirements for ReducTrain

Design requirement	Acceptance criteria
Durability	No maintenance needed before 1000 uses
Ease of assembly	< 6 h to construct the device
Cost	< US $200

### Prototyping process

After brainstorming design ideas and selecting an idea that best met the design criteria, the team quickly developed an initial prototype to get feedback and check if their understanding of the reduction process was correct. Using an academic makerspace that included 3D printers and basic woodworking tools, the team created the initial prototype using polystyrene foam, thin plywood, and rubber bands. The team made many shoulder joint models and various anterior and posterior shoulder regions out of foam, allowing for easy, rapid feedback. The team used rubber bands to simulate muscles. Even though the force needed for reduction was very low, with input from their clinical advisors, the team was able to establish the correct path of the joint as well as the approximate size. The team did not pay attention to true anatomical accuracy but rather to a more functional, feel-based accuracy.

Next, the team focused on building a stronger model that could accommodate realistic forces during a shoulder reduction. This prototype used thicker plywood as the base, a 3D-printed shoulder model, resistance bands (commonly used for exercise) to simulate the muscles, and wooden dowels as the arm. A diverse group of Duke Hospital emergency physicians, ranging from novice to highly experienced, informed the iterations during this prototyping phase. Initially, residents were not given instructions, and the team observed how they used it and what they said about its feel. Later, the residents were given verbal instructions on how to use the model, and each physician gave feedback about what should be changed, what felt realistic, and any other relevant feedback. These conversations identified multiple components in the model that still needed revision.

To improve ReducTrain, this feedback was incorporated into all aspects of the design, especially the 3D-printed assemblies. Once the design was settled, the team built the final ReducTrain. Added features included an adjustable tension system, a higher fidelity arm, standardization of the base, and the ability to cover the model with a shirt. Step-by-step instructions and photos, including a link to the 3D printable file, for how to build ReducTrain are in the Additional file 1: Appendix.

## ReducTrain description

**Fig. 1:**
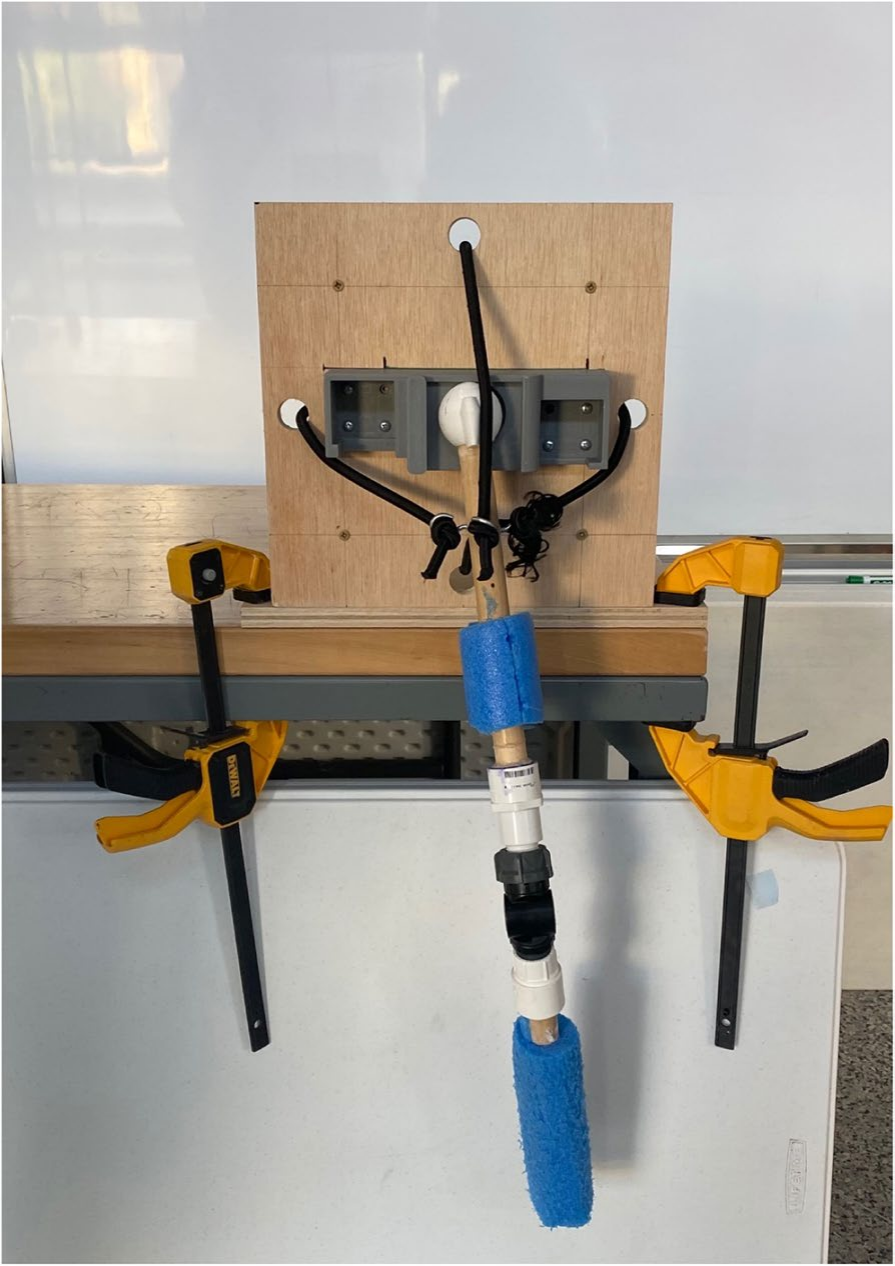
ReducTrain assembly. Arm is in the healthy (i.e., non-dislocated) position for the traction-countertraction model. ReducTrain is attached to the table with two clamps

ReducTrain is the shoulder reduction model made of wood, resistance bands, and a 3D-printed model joint and socket (Fig. 1). Key components are described in detail, along with further design rationalization.

### Support base

The base of the model is made of plywood (Fig. 1). The front, upon which a 3D-printed shoulder piece can be attached, is 13 inch (33.0 cm) × 13 inch (33.0 cm) × 0.75 inch (1.9 cm). Four holes are cut in the front plywood for resistance bands to go through to the back. The rear of the model is three more pieces of plywood glued together, each with dimensions 9 inch (22.9 cm) × 9 inch (22.9 cm) × 0.75 inch (1.9 cm). The vertical front face is attached to a 13 inch (33.0 cm) × 9 inch (22.9 cm) × 0.75 inch (1.9 cm) plywood base via two 4-inch (10.2-cm) zinc-plated heavy duty corner braces for stability. This sturdy plywood base allows the device to be clamped to the edge of any table, making the whole device quite portable.

### Shoulder

**Fig. 2:**
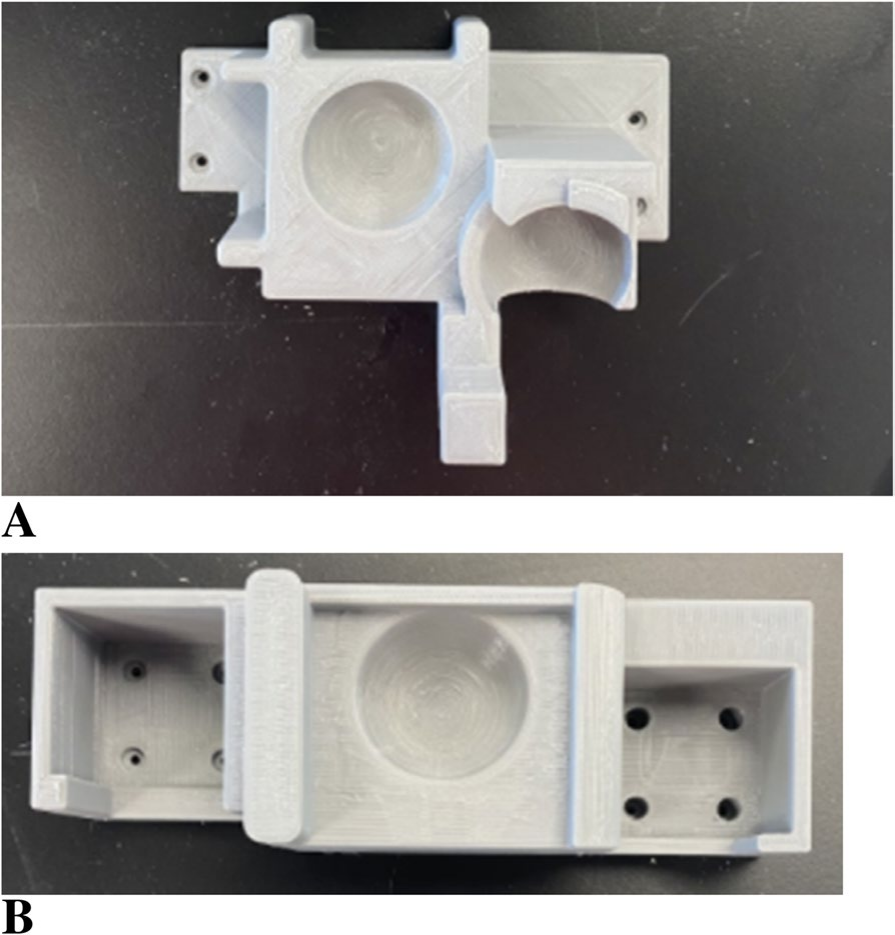
**A** The external rotation 3D-printed assembly. **B** The traction-countertraction 3D-printed assembly

Figure 2A shows the shoulder assembly for the external rotation shoulder reduction method, and Fig. 2B shows the assembly for the traction-countertraction shoulder reduction method. From left to right on each assembly, the “pockets” are an anterior dislocation, normal healthy location, and posterior dislocation. The 3D-printed assemblies are attached and interchanged to the front plywood of the model via four 1-inch (2.5 cm) screws.

Using 3D printing allowed for rapid iteration and creation of precise geometries that would have been hard to replicate using any other technology. Through feedback from our clinical advisors, the model grew to include lips, rounding, and a joint divot. Specifically, a lip protrudes on the back side of the humeral head to provide realistic feel and allows the humeral head to fit snugly into the shoulder “chambers” of the 3D-printed assemblies. The humeral head will only move out of the chamber into another one if a proper reduction motion is applied. These features were included to increase the face validity of the feeling of the device, even though they are marginally based on anatomy.

### Muscle and tendons

The muscle simulation was intended to create a composite muscle strength and feel, but not necessarily have anatomical accuracy to represent particular muscles and tendons in the shoulder. Four 3/8-inch diameter (0.95 cm) resistance bands were used to imitate muscle tension and keep the shoulder in place. An 18.5-inch (47.0 cm) band represents anterior muscles, a 20-inch (50.8 cm) band represents the posterior, a 19.5-inch (49.5 cm) band goes through the bottom, and another 19.5-inch (49.5 cm) band goes through the top. Shorter bands yield higher tensions, and each band is cut to a different length to mimic relative shoulder tension.

**Fig. 3:**
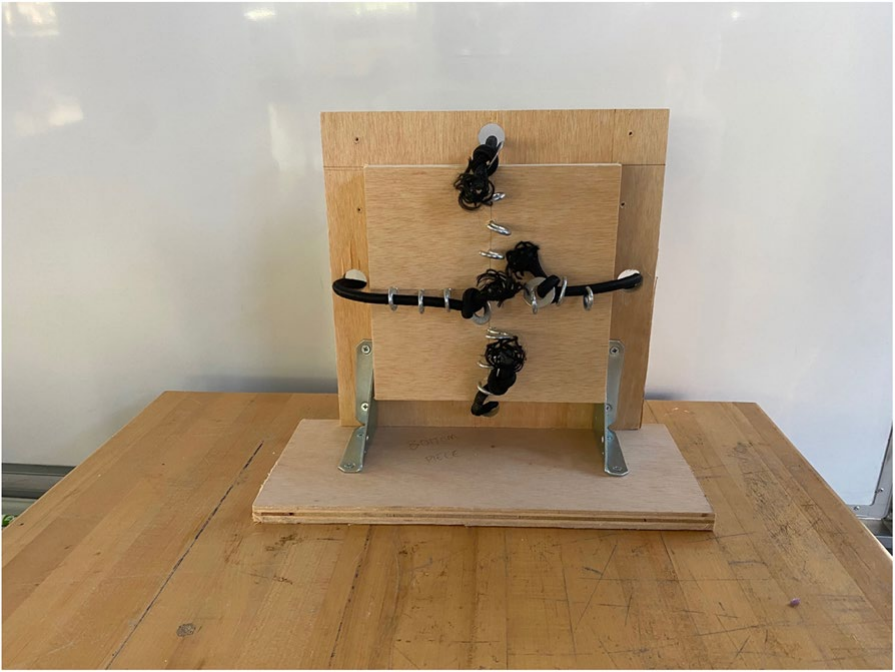
Backside of ReducTrain assembly with all four elastic cords secured through screw eyes

The resistance bands stretch around the wooden base to pull the ball joint into the socket. The bands are knotted and inserted into eye hooks on the upper half of the arm. Each band then travels through a hole drilled in the wood to the back of the model. Washers are tied into the band on the back side to prevent the bands from slipping (Fig. 3), and the knots are then attached into eye hooks on the back of the base. Attached to the back of the model is a line of eye hooks that allow for increased or decreased tension. To simulate different patient conditions and forces, the bands can be tightened or loosened, allowing for an adjustable range of tensions. For example, securing the bands to eye hooks closer to the edges of the back results in weaker resistance.

### Arm

**Fig. 4:**
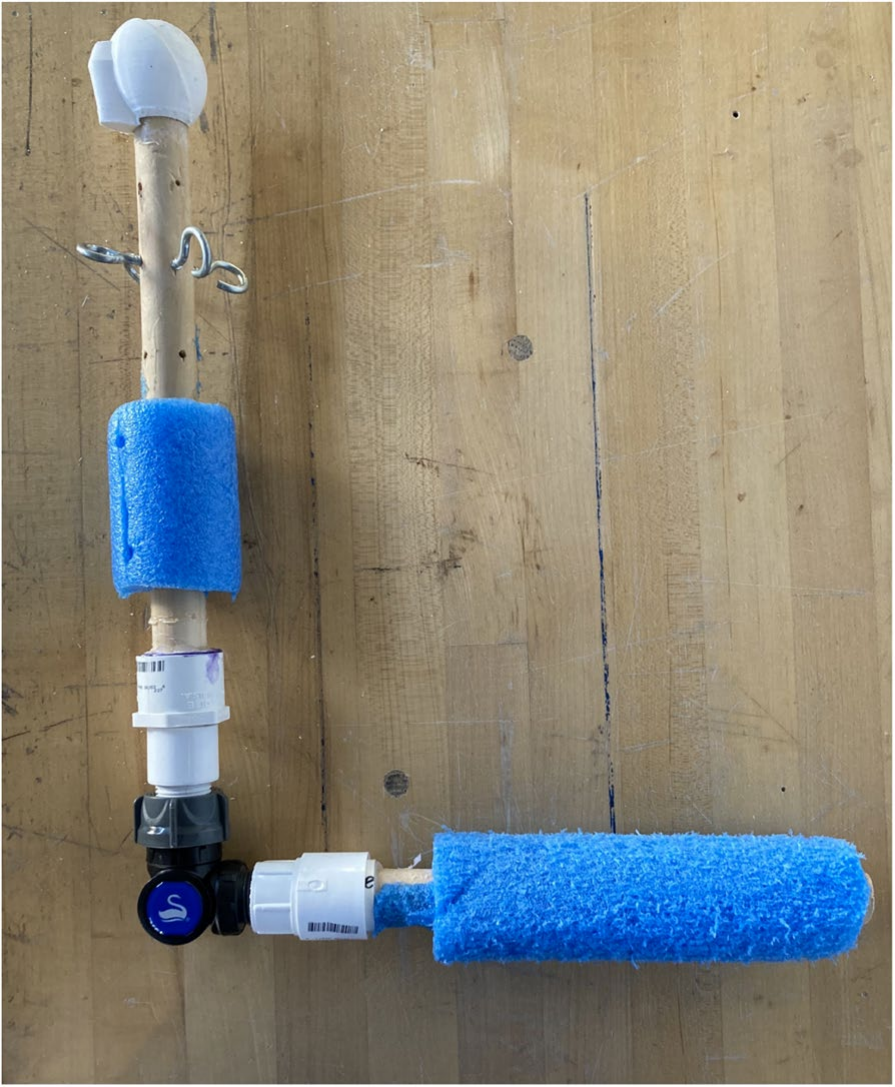
ReducTrain arm assembly that is equipped with the 3D-printed humeral head, four screw eyes for tension band securing, swivel hose pipe adaptor, and cut pool noodles

The arm component of ReducTrain consists of the 3D-printed humeral head attached to the top of a 12-inch (30.5 cm) dowel (Fig. 4). This dowel, representing the upper arm, is then connected to a 10-inch (25.4 cm) dowel that represents the lower arm. Connection of the two dowels is via PVC adapters attached to a swivel hose connector that mimics the functionality of an elbow, allowing trainees to move the upper and lower arm independently. Eye hooks are attached to the upper arm dowel for tension band attachment.

**Fig. 5:**
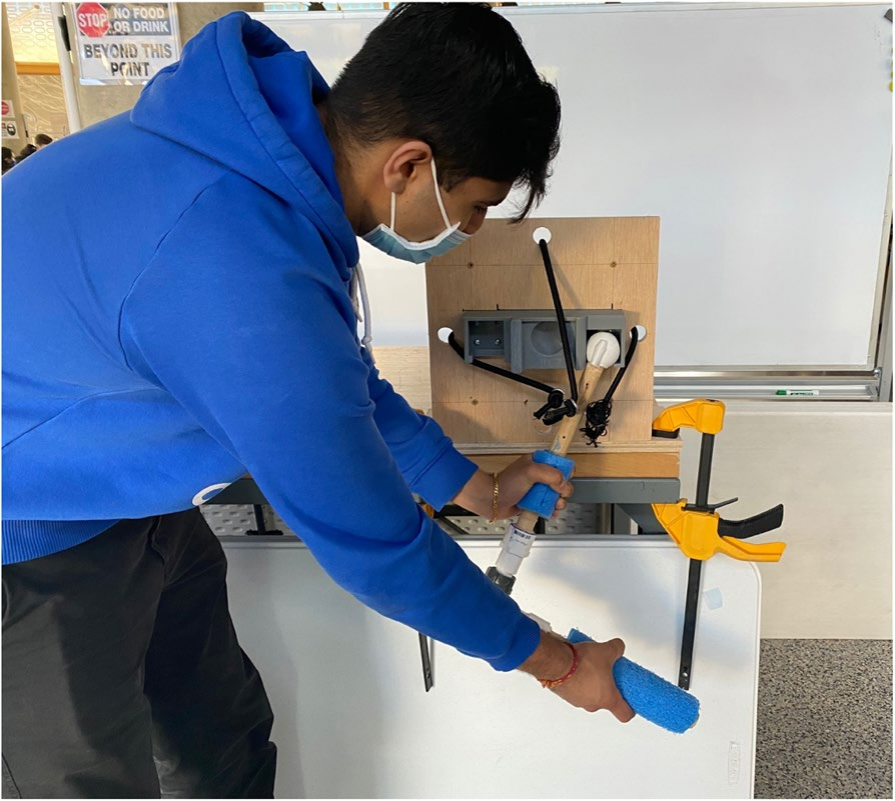
ReducTrain assembly in use. Arm is in the unhealthy (i.e., dislocated) position for the traction-countertraction model. ReducTrain is attached to the table with two clamps

A polyethylene foam cylinder was cut along its long edge and glued onto both dowel pieces to present a thicker, fleshier arm. The 3D-printed humeral head interfaces with the 3D-printed assembly (Fig. 5).

### Model covering

The model can be carried by one person and set up in many teaching spaces. To use the device for training, the bottom plywood base must be clamped down to a hard surface such as a desk or table for stability. ReducTrain can be covered with a shirt or jacket to hide the 3D-printed assemblies. The 3D-printed models have some simulated anatomy to limit range of motion but largely allow for realistic, free motion. Trainees can move the arm and humeral head from dislocated positions to normal positions and practice the reduction movement.

## ReducTrain testing

### Testing — strategy and protocols

While informal testing was conducted throughout the iterative prototyping process to guide design improvements, performance and operational testing were done on the final model against the acceptance criteria. Testing focused on durability, ease of assembly, and cost. Simulator face and content validity were outside the scope of this work and are noted as a future direction.

#### Durability

The team tested the model’s durability by performing 2000 traction-countertraction reductions and examining the model for signs of fatigue. To maintain a correct holistic resistance, bands were under different amounts of tension in the normal position. Each of the four bands stretched a unique amount, which was determined by measuring the initial length (in the normal position) and the maximum distance the bands had to stretch during a traction-countertraction reduction (Table 2, initial and final positions).

**Table 2:** Fixed displacement band testing

	At rest position (cm)	Initial position (normal position) (cm)	Final position (maximum distance) (cm)	Percentage change between initial and final position	Initial force (mean ± std.) (*N*)	Force after 1000 trials (mean ± std.) (*N*)	% force change: initial to 1000	Force after 2000 trials (mean ± std.) (*N*)	% force change: initial to 2000
Anterior	18.5	34.5	44.5	28.9%	22.4 ± 0.4	22.8 ± 0.3	+1.6%	24.9 ± 0.4	+11.2%
Posterior	20.0	35.5	43.0	21.1%	18.2 ± 0.1	20.3 ± 0.4	+11.9%	17.3 ± 0.9	−4.6%
Bottom	19.5	36.5	44.0	20.5%	20.7 ± 0.3	19.9 ± 0.2	−4.0%	19.9 ± 0.2	−3.7%
Top	19.5	36.5	43.0	17.8%	17.7 ± 0.3	15.6 ± 0.2	−11.8%	15.0 ± 0.2	−15.6%

The team inspected the entire model, including a visual inspection of the 3D-printed components to ensure no damage and a visual and tactile inspection of the model arm to ensure it still had full range of motion. To quantitatively analyze the fatigue on the resistance bands, they were tested on an Instron extensometer device using a fixed displacement test after 0, 1000, and 2000 reductions. The fixed displacement range on the Instron extensometer was equal to each band’s unique stretch distance during a reduction. Prior to repetitive reductions, the forces required for three sequential displacements were recorded and then averaged. The bands were then attached to the ReducTrain device, and 1000 reductions were performed. The bands were removed, and the same fixed displacement test on the Instron was repeated, again collecting three displacements for each band. The Instron displacement test was repeated once more after 1000 additional reductions (for 2000 total reductions) were completed.

#### Ease of assembly

Three first-year undergraduate engineering students were given the instructions and materials to build the device. First-year undergraduate engineering students were chosen to complete this test because they did not have significant prototyping experience but had an interest and some familiarity with basic tools. The team estimated that healthcare providers that would be inclined to build this device would have similar skills. They completed the build in a makerspace with the required tools while timed and received no further instructions in regards to how to build the device. Any questions or clarifications asked by the students in regards to the wording of the instructions were noted.

#### Cost

The total cost of the device was calculated by adding up the prices of all required components in the materials list. Prices were for the smallest quantity that could be purchased, so this total cost results in some extra materials. Prices for the 3D-printed components were included by adding up the cost of the PLA filament per gram needed to construct the assemblies.

### Testing results

No damage on any device component was found during visual and tactile inspection of the device after the durability test. The forces required to stretch the resistance bands after 0, 1000, and 2000 reductions are shown in Table 2. The top resistance band experienced the most force reduction, losing 15.6% of initial strength. The posterior and bottom resistance bands have force losses of less than 5% after 2000 reductions. The anterior band actually showed an increase in the force (noted as a positive change) required to stretch its fixed displacement. This increase in force could have been due to a variety of factors, including work hardening and variability in the measurement device. Overall, it was concluded that the bands do not undergo significant uniform degradation in 2000 reductions.

Excluding 3D printing times, the assembly times were 4, 3, and 3 h; thus, the average assembly time for the base and arm was 3 h and 20 min. Instructions were edited for clarity based on points of confusion noted by the students. The cost to purchase all materials was around US $190, given the sourced materials list (Additional file 1: Appendix).

## Discussion

The ReducTrain model fills a void in emergency medicine and orthopedic simulation education [8–11]. The device does not provide an anatomically identical model of the shoulder, arm, or body but does act as a strong tool for deliberate practice of shoulder reductions [5].

### ReducTrain meets tested design requirements’ acceptance criteria

The ReducTrain model met the acceptance criteria for durability, ease of assembly, and cost. The durability test indicated that the device can be used more than 1000 times without noticeable wear, thus meeting the acceptance criteria. The top band should be replaced after 2000 uses to maintain consistency of the band strength; alternatively, a different band that provides a similar and consistent force could be found. The other bands changed to a limited extent over the course of 2000 reductions. One limitation of this test was that the minimum distance of the traction-countertraction reduction was chosen; in practice, bands may stretch different and farther distances.

The ease of assembly test suggests that with the exception of the 3D-printed components, the model can be built in 3 h and 20 min with access to appropriate tools and materials. This time is comfortably under the 6-h acceptance criteria. The final devices that the participants made each had their own shortcomings (i.e., the polyethylene foam cylinder was cut too short, frame was not centered), but each of the devices both looked and properly functioned like the original ReducTrain.

The cost of US $190 accounted for all materials in the final prototype. This cost is very low compared to commercial products [9–11] of this caliber within emergency medicine and is slightly lower than our original goal of < US $200. The cost of materials may change, so it is possible that those replicating the device might spend slightly more or less money than the team did. Price could likely be driven down by selecting alternate, less expensive materials.

No systematic evaluative testing regarding simulation face validity was completed by physicians on ReducTrain once the device was finalized. The inclusion of an Institutional Review Board (IRB) approval would have allowed the team to conduct research on a wider scale and test simulation validity, along with other potential goals, in a more effective manner.

### Reflecting on prototyping

Significant design decisions were made with the goal of clinicians being able to easily recreate the simulator without the need for complex tools. With the growing prevalence of makerspaces, it was assumed that clinicians would have access to a makerspace and its tools, as well as staff who can help with construction [14]. This assumption is confirmed by work in other fields of medical simulation that use 3D printing to produce low-cost models [15, 16]. To assemble the ReducTrain, a makerspace with woodworking tools such as saws and drills is required, as well as a 3D printer. We encourage those replicating our design to seek out a makerspace so they have access to quality tools.

During the design process, the team faced several challenges. The most significant was evaluating the face validity of the “feel” of the device during iterative prototyping. Because none of the undergraduate team had conducted a shoulder reduction before, it was incredibly difficult to simulate a real reduction with an acceptable accuracy. The team had to rely on research and informal interviews with experienced healthcare providers. While the team was ultimately able to get enough feedback, delays in prototyping were experienced when waiting on clinical advisors. That being said, the initial lack of familiarity resulted in the team being very deliberate about ensuring that the range of motion was accurately limited. For example, an advisor remarked how clever it was that the team had added a wall to limit the range of motion, noting that he had completely forgotten that the shoulder did not move to that area during an external rotation reduction.

During the prototyping process, the team also faced difficulty with making the model flexible enough to accommodate other 3D-printed reduction assemblies. Because of the sequencing of the team’s prototyping steps, the team finalized the base structure and position of the bands before creating the external rotation assembly. This unintentionally limited the space available for a 3D-printed assembly and consequently constrained the reduction methods that could be easily attached. This required a reworking of the base model to accommodate additional 3D-printed assemblies.

### Use in a teaching environment

The team made ReducTrain for a teaching environment. Because the model can be used with all parts visible, it can act as a nonanatomical “map,” before acting as a physical and tactical simulator. It is important to reinforce that the features on the model are not anatomical features but instead an estimation of where the joint has to move to be reduced successfully. If this is not stressed, there is a risk of negative learning, where trainees may learn incorrect information. As proficiency improves, the assembly can be covered with a large shirt or sweatshirt. This covers the anatomy, so the user has to reduce the joint purely by feel. This is also more realistic and allows a user to progress in training.

Because the model is open and large, it is easy to group around and learn in collaborative training settings. From a pedagogical perspective, there are many potential scenarios for using the device. The three observed by the design team include the following:

1. Using the device, one clinician illustrated the reduction path and its anatomical landmarks. He then helped a colleague guide the humeral head on her first try, so she could get a feel for it, and then, she tried herself.
2. Two peers who were familiar with reductions showed each different reduction techniques that the other was not familiar with, including scapular manipulation and the Milch technique. Note that the model was not designed to accommodate these reduction techniques, so this may carry the risk of negative learning. Though they felt that certain techniques did not feel similar to manipulations on patients, they found ReducTrain to be a useful platform to discuss the theory because they could reference and move the arm and body. It is important to note the model should not be used to instruct other reduction techniques as it does not replicate the procedure accurately, but it did assist them in explaining the movement they were describing.
3. An experienced clinician presented his three favorite techniques to a group of four residents who had limited or no experience. Two of these techniques were reductions that the model was intended for, while the other one was not. While presenting, he adjusted the tension to simulate different patients. The group of four then experimented one reduction at a time — replicating the techniques.

These exchanges illustrate the power of the ReducTrain at its core: conversation and repeated practice should lead to improved performance in an authentic medical situation. This practice is especially critical for shoulder dislocations, given the considerable force required and the nuanced movements of the reduction [3].

### Adjustable features simulates range of conditions

By making the 3D-printed assemblies, base wooden model, and instructions all open source, the team invites others to collaborate on ReducTrain. The 3D models are accessible online at the links included in the Additional file 1: Appendix, and comments and downloads can be performed at the Thingiverse link. The 3D-printed shoulder assembly has three main benefits for end users. First, the 3D-printed assembly allowed for easy refinement of the existing 3D design files and for the development of different joint assemblies. Other users could make their own assemblies to expand the usability to other reductions, and there is space to allow for both posterior and anterior dislocation regions. These new assemblies can be attached to the base model. Second, the design allowed for broken assemblies to be replaced easily and at a low cost. Third, 3D printing allows for a high precision simulator to be created with access to limited tools and materials.

The screw eyes allow for the tension of the resistance bands to be increased or decreased based on which screw eye the band is hooked. This simulates different patients, as different patients require more or less force to be applied during the reduction procedure. Adjustable strength also allows for initial training to occur on an easy to use, low force model. As trainees are learning shoulder reductions, the force can be increased, making nuanced motions far more difficult. Several parts of the design, including the arm and resistance bands, can also be interchanged or replaced.

### Limitations of shoulder reduction model and future work

Embedded in the goals of this project — namely to create a low-cost, easily replicated, and simple simulator — are its limitations. The trade-offs inherent in the engineering design process sacrificed an exact anatomical representation of the human body for a more simplified design [17]. For example, the geometry of the 3D-printed assemblies was finalized based on informal feedback from experienced clinical staff at Duke Hospital, not on attempts to anatomically replicate the shoulder. These trade-offs are made frequently in other low-cost simulators [18, 19].

Additional limitations to the model include the following: (1) no form of muscle and soft tissue in the shoulder, (2) a highly simplified arm, (3) simplified tendon structure, (4) no skin-like material covering any part of the model, and (5) limited auditory feedback. The model arm weighs less and is smaller than a typical human arm. While a more anatomically accurate elbow joint and realistic flesh and muscle system could have been created, the current version has a realistic range of motion and a durable design. Added features would have severely complicated the ability for a wide range of clinicians to replicate the design.

Additionally, the use of the ReducTrain in a training setting must be monitored to avoid risk of negative learning [3, 5]. While it can be used as a reference tool, training should be conducted under the supervision of an experienced instructor to avoid misunderstanding or misrepresentation.

Construction of the ReducTrain may be difficult in some contexts as well. For healthcare providers who have very limited experience using tools, the time to build ReducTrain may exceed 4 h. Additionally, without access to a 3D printer, a provider might need to outsource the printing of the 3D assemblies to online printing services which may make initial construction and repairs more cumbersome and costly. Finally, in a low-income setting where 3D printing may not be available, an assembly made from clay or another material may need to be substituted. If materials are not readily available, substitutions may be needed. While these changes may reduce precision and accuracy, many of the components would remain functional.

Future work includes an IRB-authorized study to evaluate the simulation face validity of ReducTrain. While out of the scope of this project, involving many experienced physicians in a carefully crafted study on the face validity of the simulator would further verify the legitimacy of the model and might lead to further refinements. Moreover, future versions of the model could include a wider variety of 3D-printed frames to account for a greater number of reduction techniques. The materials required to make the device alongside the instructions could also be placed in a kit that could be produced and sold en masse, rather than having those who wish to remake the device go to a makerspace.

## Conclusion

The ReducTrain can support the instruction and deliberate practice for medical staff as they learn shoulder reduction methods. In particular, ReducTrain models the traction-countertraction and external rotation reduction methods. Based on testing, the device should last for more than 2000 uses, cost less than US $200, and be able to be built in 3–4 h. Using tools and materials commonly found in a makerspace and the instructions provided, emergency departments and other teaching units can build the shoulder reduction model to support the education of their medical staff.

## Supplementary Information

**Additional file 1: Appendix.** Part 1: Preparing the wood. Part 2: Assembling the body. Part 3: Assembling the arm. Part 4: Assembling the bands. Part 5: Final assembly. Part 6: Using the device. **Fig. A1.** The 3D printed humeral head. **Fig. A2.** The external rotation 3D printed assembly. **Fig. A3.** The traction-countertraction 3D printed assembly. **Fig. A4.** The front plate with the holes and their locations labeled. **Fig. A5.** Alignment line for the traction-countertraction assembly. **Fig. A6.** The traction-countertraction assembly with its alignment line on the top edge of the assembly. **Fig. A7.** Alignment line for the external rotation assembly. **Fig. A8.** Standard (left) and “opened” (right) screw eyes. **Fig. A9.** The location of the hook holes. **Fig. A10.** The back of the wooden assembly with the arrangement of screw eyes, base plate attached, and triangle brackets secured. The front plate is centered and flush with the base plate so that there is 1.5 inch (3.81 m) on either side. **Fig. A11.** Side views of the image shown in Figs. 1 and 3. The triangle brackets are in line with the back plates, which are aligned and glued together. **Fig. A12.** The completed top half of the arm. **Fig. A13.** Transition showing adapters before and after screwing on to the swivel hose pipe adapter. **Fig. A14.** Applying hot glue to the ends of the dowels serves to create a tight fit that adheres well to the PVC connectors. **Fig. A15.** The dowel is pressed firmly into the connector in order to create a tight fit, with the hot glue filling the gap. **Fig. A16.** The sliced open pool noodle is wrapped around the dowel to simulate flesh. **Fig. A17.** The final arm assembly. **Fig. A18.** The elastic cords with the washers tied on. **Fig. A19.** The external rotation assembly attached to the final body. **Fig. A20.** Back side of ReducTrain assembly with all four elastic cords secured through screw eyes. **Fig. A21.** Front side of device with resistance bands through the holes on the edge and then attached to screw eyes on the arm. **Fig. A22.** The correct set up of the ReducTrain with clamps. **Fig. A23.** The humeral head in the anterior dislocation position of the traction-countertraction assembly. **Fig. A24.** Attempting to manipulate the humeral head to the healthy position. **Fig. A25.** The large sweatshirt over the model with a mannequin head on top to make the model look more realistic [20–37].

## Data Availability

Not applicable
