# Supplementary material for: Simulation device for shoulder reductions: overview of prototyping, testing, and design instructions
Source: Adv Simul (Lond). 2023 Mar 9;8:8. doi: 10.1186/s41077-023-00246-3 (PMC9999631; doi:10.1186/s41077-023-00246-3)
Supplement: Supplementary file 1 — Additional file 1: Appendix. Part 1: Preparing the wood. Part 2: Assembling the body. Part 3: Assembling the arm. Part 4: Assembling the bands. Part 5: Final assembly. Part 6: Using the device. Fig. A1. The 3D printed humeral head. Fig. A2. The external rotation 3D printed assembly. Fig. A3. The traction-countertraction 3D printed assembly. Fig. A4. The front plate with the holes and their locations labeled. Fig. A5. Alignment line for the traction-countertraction assembly. Fig. A6. The traction-countertraction assembly with its alignment line on the top edge of the assembly. Fig. A7. Alignment line for the external rotation assembly. Fig. A8. Standard (left) and “opened” (right) screw eyes. Fig. A9. The location of the hook holes. Fig. A10. The back of the wooden assembly with the arrangement of screw eyes, base plate attached, and triangle brackets secured. The front plate is centered and flush with the base plate so that there is 1.5 inch (3.81 m) on either side. Fig. A11. Side views of the image shown in Figs. 1 and 3. The triangle brackets are in line with the back plates, which are aligned and glued together. Fig. A12. The completed top half of the arm. Fig. A13. Transition showing adapters before and after screwing on to the swivel hose pipe adapter. Fig. A14. Applying hot glue to the ends of the dowels serves to create a tight fit that adheres well to the PVC connectors. Fig. A15. The dowel is pressed firmly into the connector in order to create a tight fit, with the hot glue filling the gap. Fig. A16. The sliced open pool noodle is wrapped around the dowel to simulate flesh. Fig. A17. The final arm assembly. Fig. A18. The elastic cords with the washers tied on. Fig. A19. The external rotation assembly attached to the final body. Fig. A20. Back side of ReducTrain assembly with all four elastic cords secured through screw eyes. Fig. A21. Front side of device with resistance bands through the holes on the edge and then attached to screw eyes on the arm. Fig. A [file 41077_2023_246_MOESM1_ESM.zip › APPENDIX v3_ESM.docx]

**APPENDIX**

The required materials and tools are noted below. The custom CAD files of the humeral head and shoulder assemblies are available on Thingiverse. Step-by-step instructions are given for assembly of a ReducTrain. Units of all numbers in the figures are inches, with metric equivalents in parenthesis.

*Required Materials:*

- 2 ft x 4 ft x ¾ inch (61.0 cm x 121.9 cm x 1.9 cm) sheet of plywood [20]
- 12 zinc lag screw eyes (3/16 inch (4.8 mm) diameter x 1 15/16 inch (4.9 cm) length x 9/16 inch (1.4 cm) internal diameter) [21]
- Two 4-inch (10.2 cm) zinc-plated heavy duty corner braces [22]
- One black and blue swan multipurpose hose swivel [23]
- Four 2.5-inch (6.4 cm) #8 wood flat head screws [24]
- Eight 1-inch (2.5 cm) #8 wood flat head screws [24]
- One 1-inch (2.5 cm) diameter round dowel (2 ft (61.0 cm) in length) [25]
- One polyethylene foam cylinder (sold as “pool noodle”) with a central hole [26]
- One 10-ft (304.8 cm) pack of ⅜-inch (9.5 mm) diameter resistance bands [27]
- Wood glue [28]
- Hot glue [29]
- Hot glue gun [30]
- Two clamps, with a clamping size greater than 4 inch (10.2 cm) for use while simulating [31]
- One ¾-inch (1.9 cm) female hose to ¾-inch (1.9 cm) male pipe PVC adapter [32]
- One ¾-inch (1.9 cm) female pipe to ¾-inch (1.9 cm) male hose PVC adapter [33]
- Humeral Head (Fig. A1), ~18 g PLA filament [34, 35]
- External Rotation Assembly (Fig. A2), ~168 g PLA filament [35, 36]
- Traction-Countertraction Assembly (Fig. A3), ~233 g PLA filament [35, 37]

*Required Tools:*

- Circular saw, hand saw, table saw, or jigsaw to cut plywood
- Jigsaw or Forstner Bit
- Drill or driver set with bits
- Tape measure
- Clamps and a workspace for holding glued pieces together
- Vice

*Construction Process:*

**PART 1: PREPARING THE WOOD**

1. Cut out one 13 inch (33.0 cm) x 13 inch (33.0 cm) front plate, three 9 inch (22.9 cm) x 9 inch (22.9 cm) backplates, and one 13 inch (33.0 cm) x 9 inch (22.9 cm) base plate.
2. Cut out the four resistance band squares on the **front plate** using either a jigsaw or a Forstner bit as shown in Fig. A4. If you have access to a Forstner bit, you can use a 1-inch (2.5 cm) diameter bit to drill circles in the locations instead of squares. Each hole should have an edge ½ inch (1.3 cm) from the edge of the front plate.
3. Draw a 9-inch (22.9 cm) bold line on the **front plate** indicated in Fig. A5.
4. Line up the traction-countertraction assembly top edge and the left and right edges with the endpoints of the 9-inch (22.9 cm) line as shown in Fig. A6. Press a drill bit firmly into the wood through the eight screw holes in the assembly to mark the hole locations.
5. Use a ⅛-inch (3.2 mm) drill bit to completely drill through the **front plate** in the hole locations marked in step 4. These holes will be used to secure the assemblies during reductions.
6. Mark the endpoints of a 6.5-inch (16.5 cm) line on the previous alignment line on the front plate as shown in Fig. A7.
7. Repeat the hole marking and drilling steps with the external rotation assembly.

**PART 2: ASSEMBLING THE BODY**

1. Glue the three **back plates** on top of each other using wood glue between each layer and clamp them together. Let them dry.
2. Clamp a screw eye into a vice. Use pliers to open the screw eyes so that the resistance bands can easily be pressed into the circle of the screw eye, but not fall out on its own (Fig. A8). Repeat for all 12 screw eyes.
3. Once dry, use a ⅛-inch (3.2 mm) drill bit to drill 12 holes in the back of the glued together **back plates**, as shown in Fig. A9.
4. Glue and clamp the three glued **back plates** to the **front face plate**. Make sure the back plates are centered, 2 inch (5.1 cm) from each edge, as seen in Fig. A10. Secure this with the four 2½-inch (1.3 cm) wood screws, drilled in the corners into the **front face plate**. The screws should be 3 inch (7.6 cm) from each corner of the **front face plate**.
5. Screw the 12 opened screw eyes into the drilled holes, aligning each set of three holes together so that the openings are placed along a straight line (Fig. A10).
6. Using the triangle bracket kit, screw the triangle brackets, 1 inch (2.5 cm) from the side edge of the **front face plate** as seen in Fig. 10.
7. Apply wood glue to the top edge of the **base plate**, then place the **front face** (which has the **back plate** glued to it) assembly on top, centered (1.5 inch (3.8 cm) from each side) so that the **front face** and long edge of the **base plate** are flush. Make sure the line you drew in step 3 of “Preparing the Wood” is on the top half of the model when the base plate is on a table, so that the 3D assemblies are in the correct position. Screw the remaining two screws from the triangle bracket into the base plate. Let dry. Left and right side views of the updated prototype to this step are shown in Fig. A11.

**PART 3: ASSEMBLING THE ARM**

1. Cut one 12-inch (30.5 cm) piece and one 10-inch (25.4 cm) piece from the wooden dowel.
2. Glue the humeral head to one end of the 12-inch (30.5 cm) piece.
3. On the 12-inch (30.5 cm) length, pre-drill four holes 3 inch (7.6 cm) below the base of the humeral head, equally spaced from each other around the dowel, as seen in Fig. A12.
4. Open four more screw eyes, using step 2 of “Part 2: Assembling the Body.”
5. Screw screw eyes into the holes so the arm looks like Fig. A12.
6. Attach and twist both PVC adapters into their respective ends of the swivel hose piece, as in Fig. A13.
7. Apply hot glue to each of the dowels and insert the dowel firmly inside the PVC connectors (Fig. A14, Fig. A15).
8. Cut one 9-inch (22.9 cm) and one 4.5-inch (11.4 cm) lengths of the polyethylene foam cylinder. Cut each open along its long edge as in Fig. A16.
9. Apply super glue to the dowel, open the polyethylene foam cylinder, and wrap the dowel in the cylinder (Fig. A16). The 9-inch (22.9 cm) section of polyethylene foam cylinder goes on the 10-inch (25.4 cm) dowel, and the 4.5-inch (11.4 cm) section of polyethylene foam cylinder goes on the 12-inch (30.5 cm) dowel. (See Fig. A17 for approximate positioning.) The final arm is shown in Fig. A17.

**PART 4: ASSEMBLING THE BANDS**

1. Cut the resistance bands into four lengths: 18.5 inch (47.0 cm) (anterior side), 20 inch (50.8 cm) (posterior side), 19.5 inch (49.5 cm) (bottom), and 19.5 inch (49.5 cm) (top).
2. Loop 1.5 inch (3.8 cm) of each resistance band through the washer, and keeping this length as fixed as possible, create a knot around the washer (so the washer is inside the knot) as in Fig. A18. Try to maintain a fixed length of band above the knot.
3. Repeat this step by knotting 1.5 inch (3.8 cm) of each resistance band on its other end, but without adding a washer as in Fig. A18.

**PART 5: FINAL ASSEMBLY**

1. Align either of the 3D assemblies to its respective holes and attach with 1-inch (2.5 cm) screws as seen in Fig. A19. Note that the alignment line should be at the top edge of the assembly.
2. Press the resistance band into the eye of the desired eye hook at the back of the device, as shown in Fig. A20. Make sure to use the correct length band in the correct positions: 18.5 inch (47.0 cm) for anterior side, 20 inch (50.8 cm) for posterior side, 19.5 inch (49.5 cm) for bottom, 19.5 inch (49.5 cm) for top. Attaching bands to screw eyes closer to the center will yield stronger tensions.
3. Pull each resistance band through a hole on the edge. Attach the free resistance band end to screw eyes on the device's arm, as shown in Fig. A21.

**PART 6: USING THE DEVICE**

1. Line up the edge of the ReducTrain flush with the edge of the table.
2. Clamp both corners of the ReducTrain to the table using 4-inch (10.2 cm) clamps (Fig. A22).
3. Select the tension setting for the bands by pulling the washer to the correct screw eye on the back of the device. Pressing the resistance bands into screw eyes closer to the center of the face plate results in higher tension.
4. Move the humeral head to the dislocated position (Fig. A23). For the traction-countertraction assembly, there is a posterior and an anterior dislocated position. For the external rotation assembly, there is only an anterior dislocated position.
5. By manipulating the arm, reduce the dislocation into its normal position (Fig. A24).
6. Place a large shirt/sweatshirt over the model to increase the difficulty of the reduction (Fig. A25).
