# Supplementary material for: Simulation device for shoulder reductions: overview of prototyping, testing, and design instructions
Source: Adv Simul (Lond). 2023 Mar 9;8:8. doi: 10.1186/s41077-023-00246-3 (PMC9999631; doi:10.1186/s41077-023-00246-3)
Supplement: Supplementary file 1 — Additional file 1: Appendix. Part 1: Preparing the wood. Part 2: Assembling the body. Part 3: Assembling the arm. Part 4: Assembling the bands. Part 5: Final assembly. Part 6: Using the device. Fig. A1. The 3D printed humeral head. Fig. A2. The external rotation 3D printed assembly. Fig. A3. The traction-countertraction 3D printed assembly. Fig. A4. The front plate with the holes and their locations labeled. Fig. A5. Alignment line for the traction-countertraction assembly. Fig. A6. The traction-countertraction assembly with its alignment line on the top edge of the assembly. Fig. A7. Alignment line for the external rotation assembly. Fig. A8. Standard (left) and “opened” (right) screw eyes. Fig. A9. The location of the hook holes. Fig. A10. The back of the wooden assembly with the arrangement of screw eyes, base plate attached, and triangle brackets secured. The front plate is centered and flush with the base plate so that there is 1.5 inch (3.81 m) on either side. Fig. A11. Side views of the image shown in Figs. 1 and 3. The triangle brackets are in line with the back plates, which are aligned and glued together. Fig. A12. The completed top half of the arm. Fig. A13. Transition showing adapters before and after screwing on to the swivel hose pipe adapter. Fig. A14. Applying hot glue to the ends of the dowels serves to create a tight fit that adheres well to the PVC connectors. Fig. A15. The dowel is pressed firmly into the connector in order to create a tight fit, with the hot glue filling the gap. Fig. A16. The sliced open pool noodle is wrapped around the dowel to simulate flesh. Fig. A17. The final arm assembly. Fig. A18. The elastic cords with the washers tied on. Fig. A19. The external rotation assembly attached to the final body. Fig. A20. Back side of ReducTrain assembly with all four elastic cords secured through screw eyes. Fig. A21. Front side of device with resistance bands through the holes on the edge and then attached to screw eyes on the arm. Fig. A [file 41077_2023_246_MOESM1_ESM.zip › Manuscript (Figures) EDITEDv3_ESM.docx]

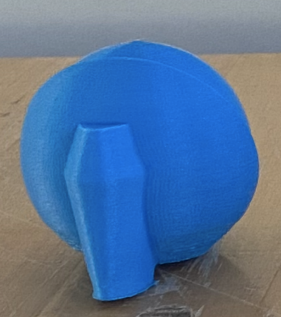


**Fig. A1**: The 3D printed humeral head.


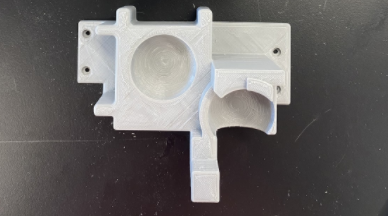


**Fig. A2**: The external rotation 3D printed assembly.


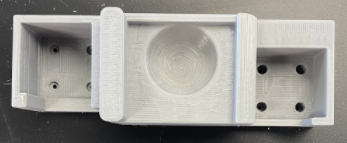


**Fig. A3**: The traction-countertraction 3D printed assembly.


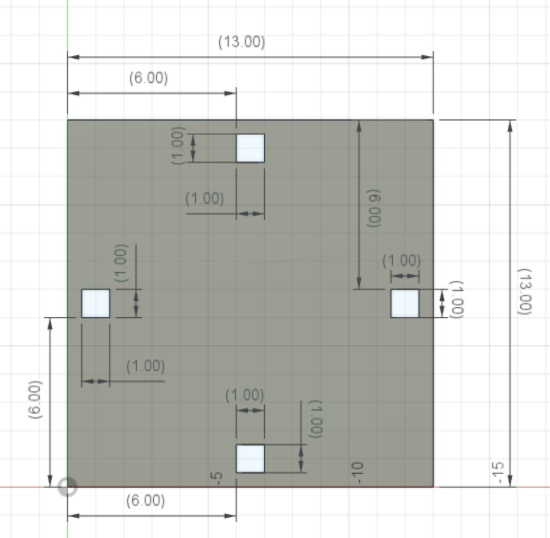


**Fig. A4:** The front plate with the holes and their locations labeled.


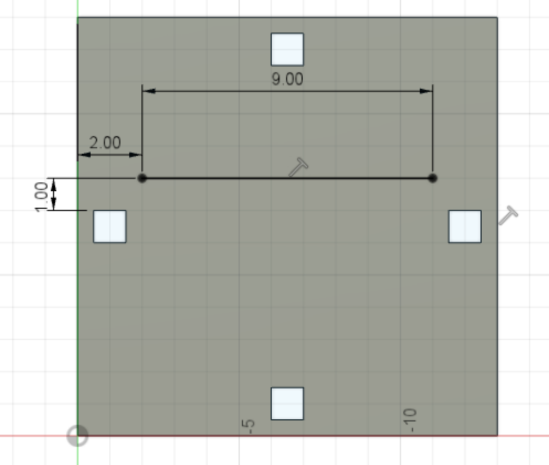


**Fig. A5:** Alignment line for the traction-countertraction assembly.

**
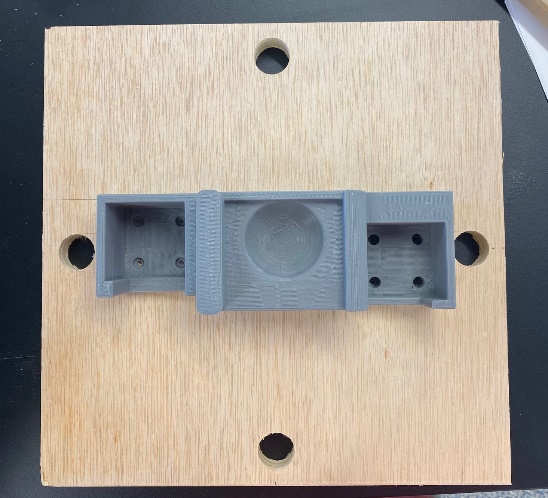
**

**Fig. A6:** The traction-countertraction assembly with its alignment line on the top edge of the assembly.


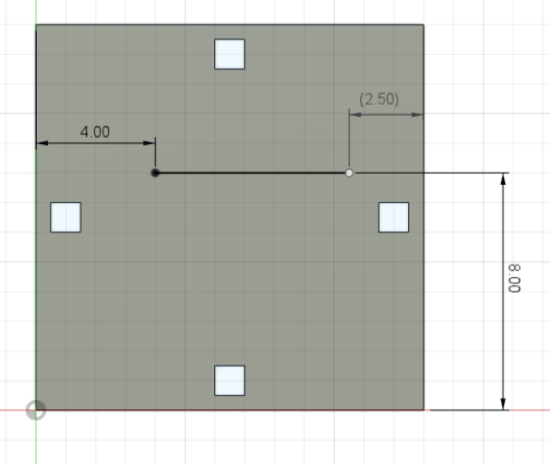


**Fig. A7:** Alignment line for the external rotation assembly.


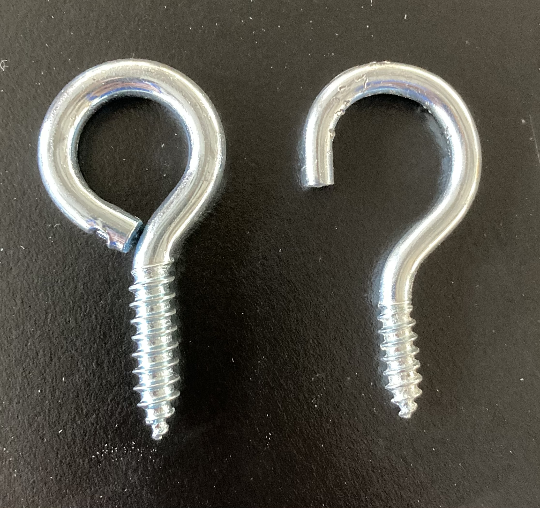


**Fig. A8:** Standard (left) and “opened” (right) screw eyes.


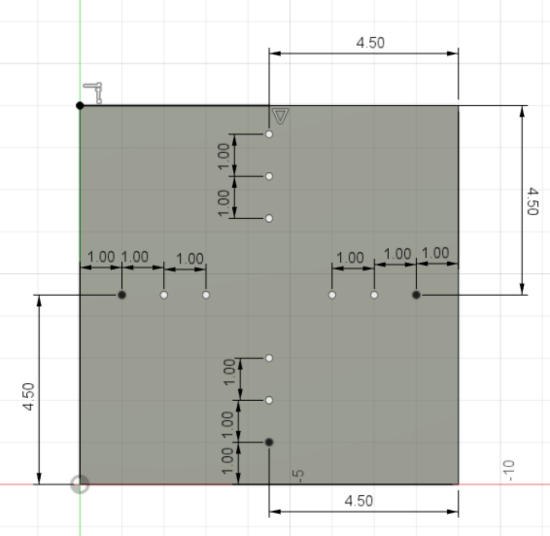


**Fig. A9:** The location of the hook holes.


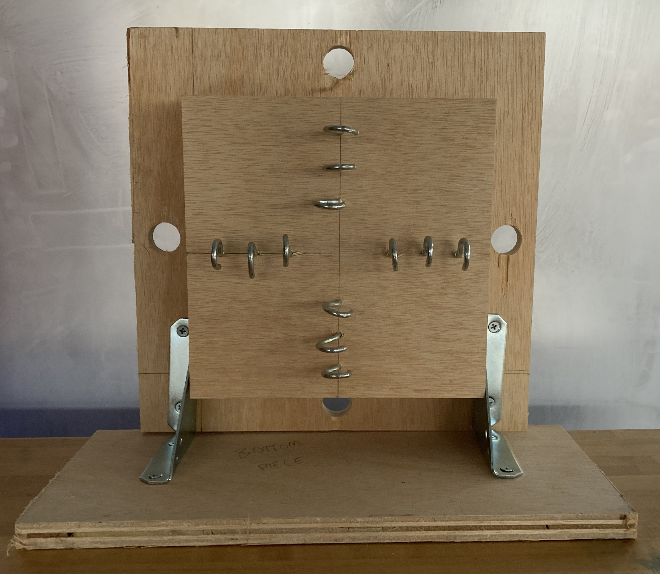


**Fig. A10:** The back of the wooden assembly with the arrangement of screw eyes, base plate attached, and triangle brackets secured. The front plate is centered and flush with the base plate so that there is 1.5 inch (3.81 cm) on either side.


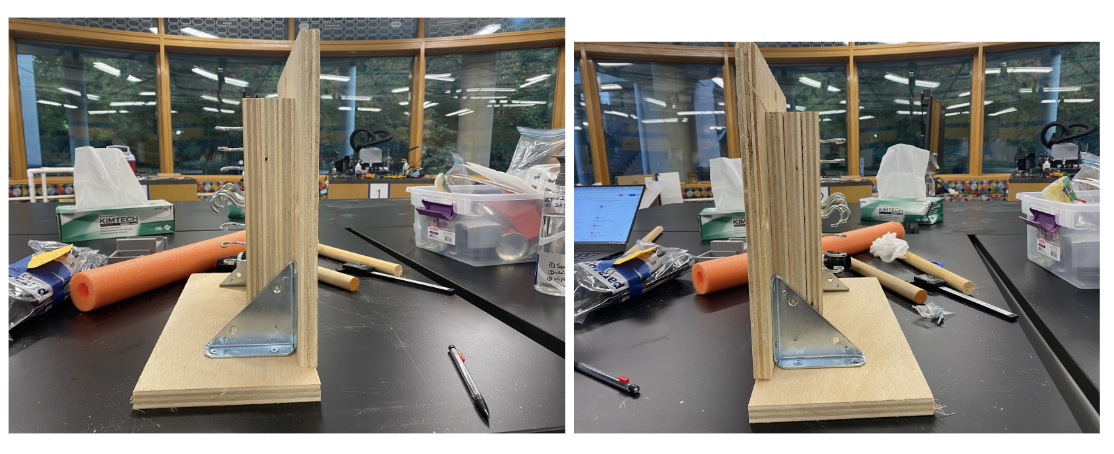


**Fig. A11:** Side views of the image shown in Figures 1 and 3. The triangle brackets are in line with the back plates, which are aligned and glued together.


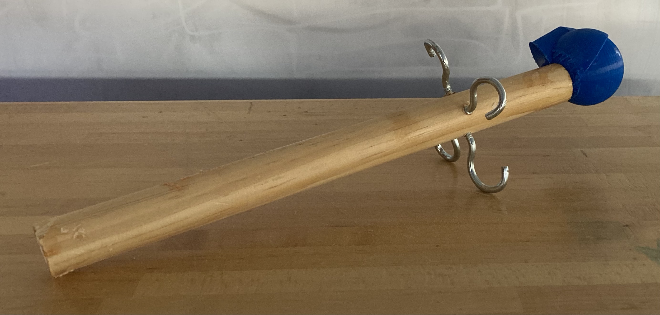


**Fig. A12:** The completed top half of the arm.


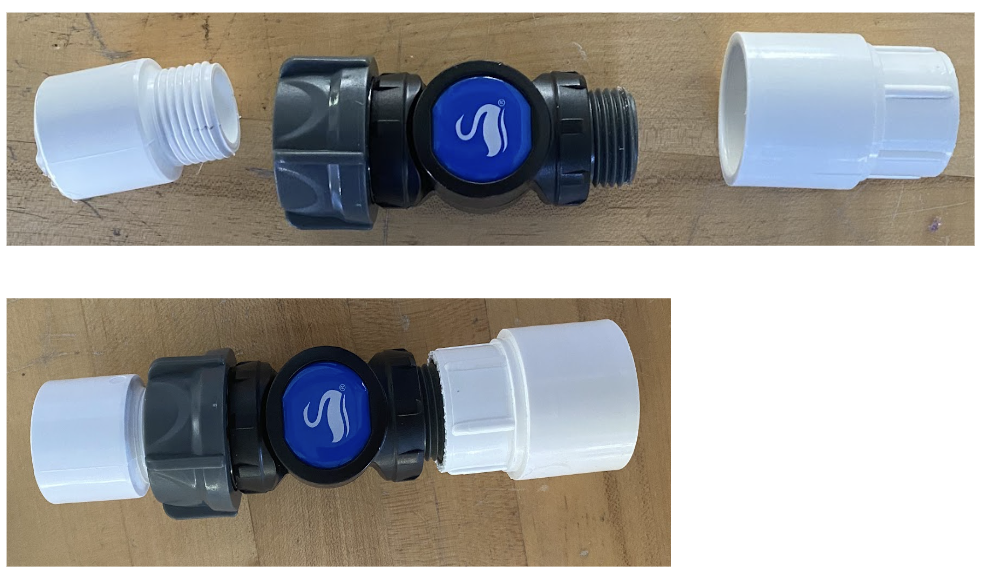


**Fig. A13**: Transition showing adapters before and after screwing on to the swivel hose pipe adapter.


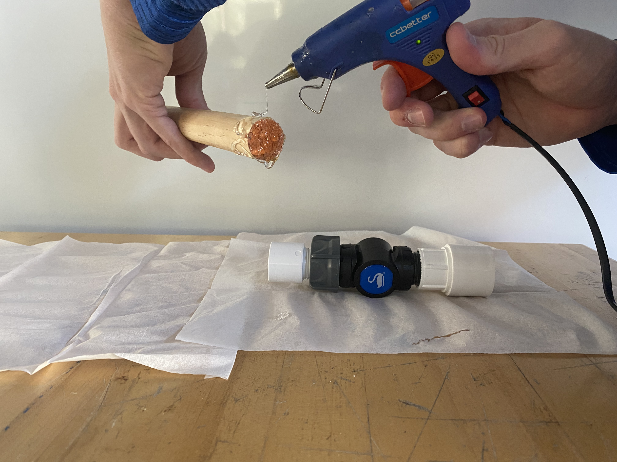


**Fig. A14**: Applying hot glue to the ends of the dowels serves to create a tight fit that adheres well to the PVC connectors.


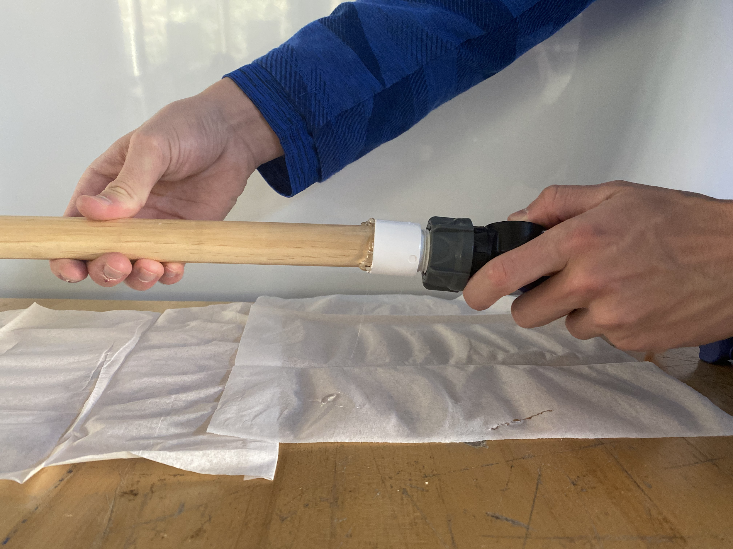


**Fig. A15**: The dowel is pressed firmly into the connector in order to create a tight fit, with the hot glue filling the gap.


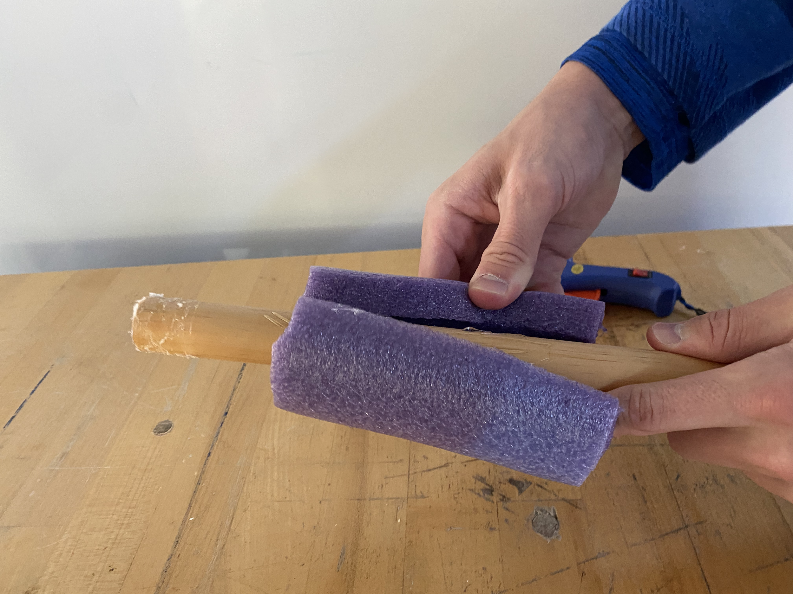


**Fig. A16**: The sliced open pool noodle is wrapped around the dowel to simulate flesh.


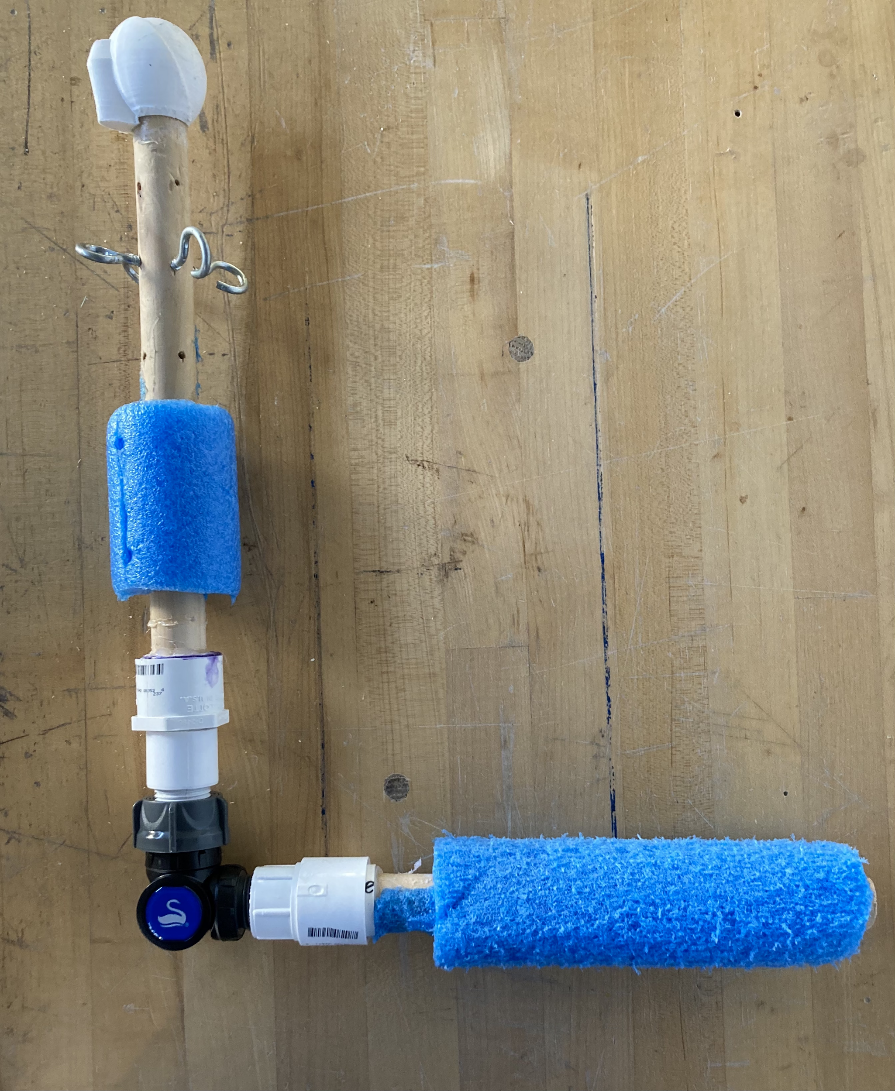


**Fig. A17:** The final arm assembly.


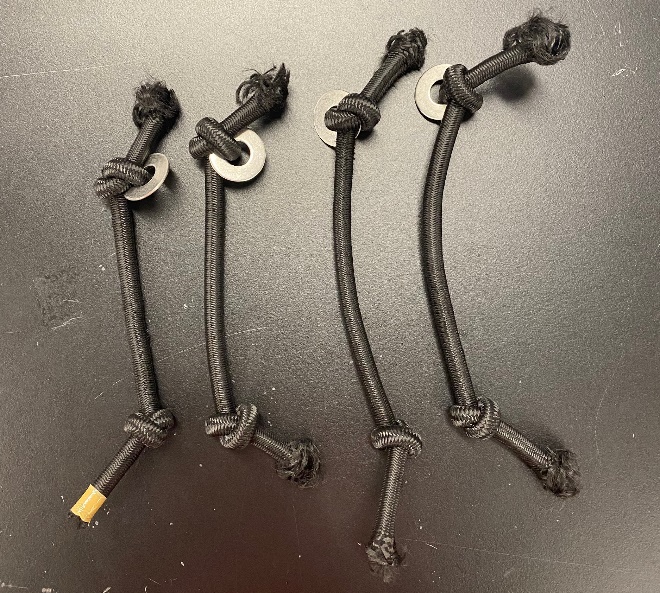


**Fig. A18:** The elastic cords with the washers tied on.


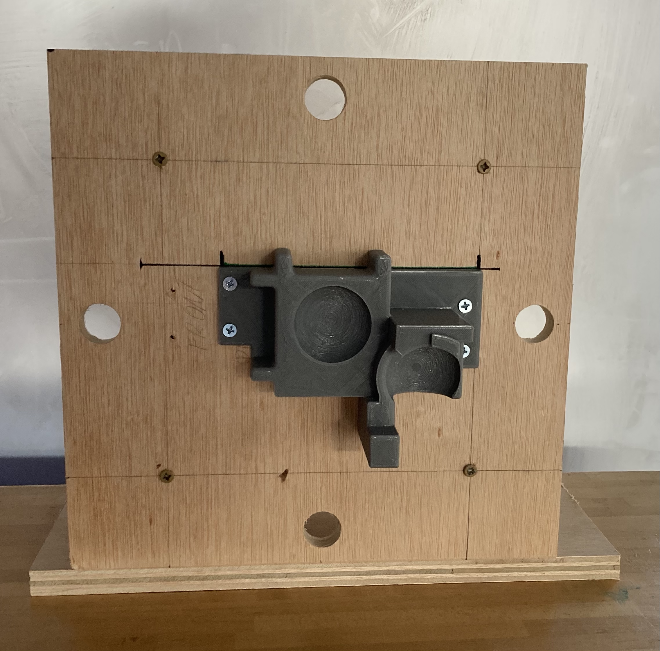


**Fig. A19:** The external rotation assembly attached to the final body.


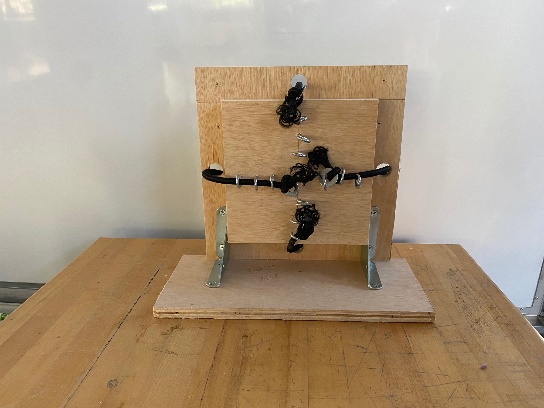


**Fig. A20**: Back side of ReducTrain assembly with all four elastic cords secured through screw eyes.


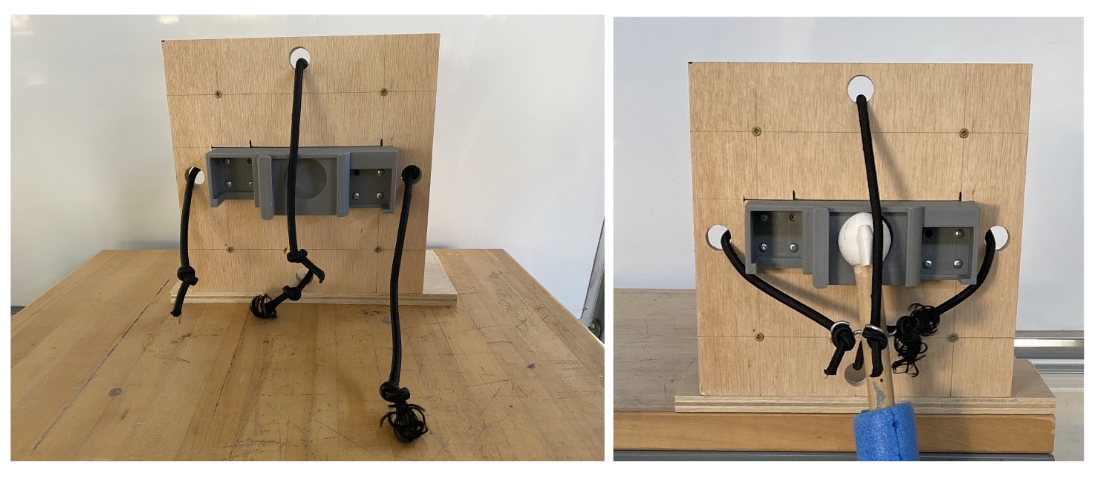


**Fig. A21:** Front side of device with bungees through the holes on the edge and then attached to screw eyes on the arm.


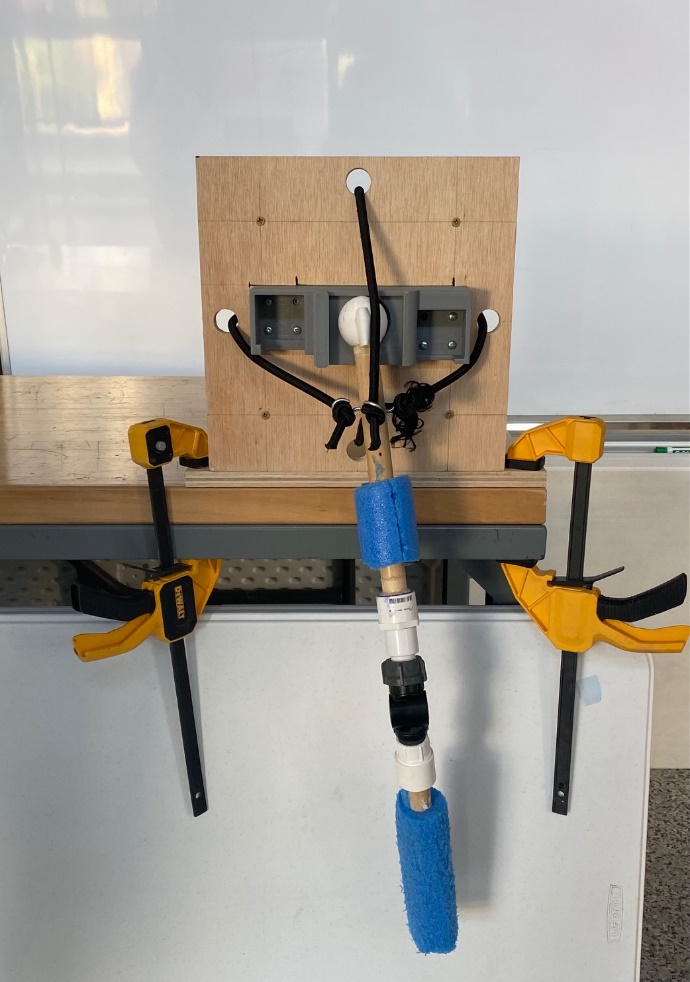


**Fig. A22**: The correct set up of the ReducTrain with clamps.


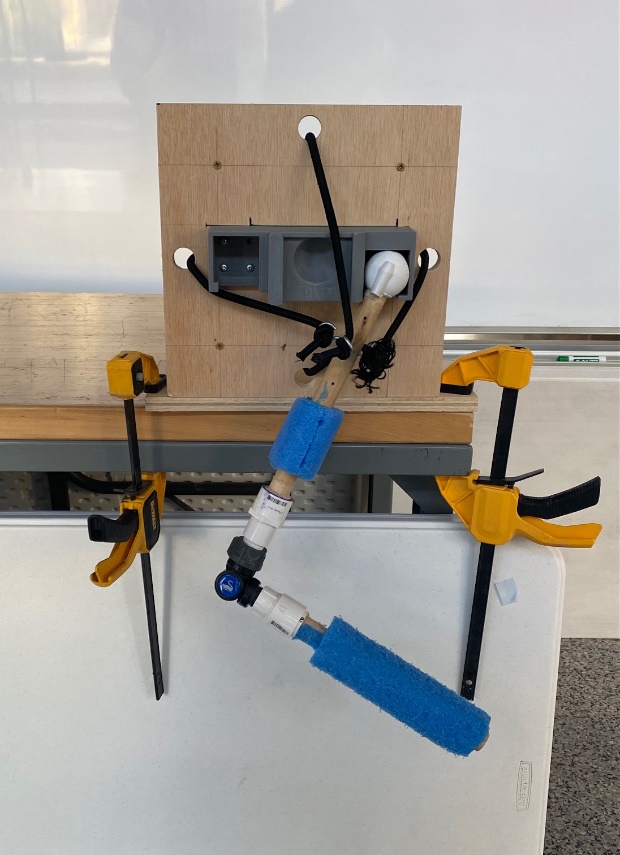


**Fig. A23**: The humeral head in the anterior dislocation position of the traction-countertraction assembly.


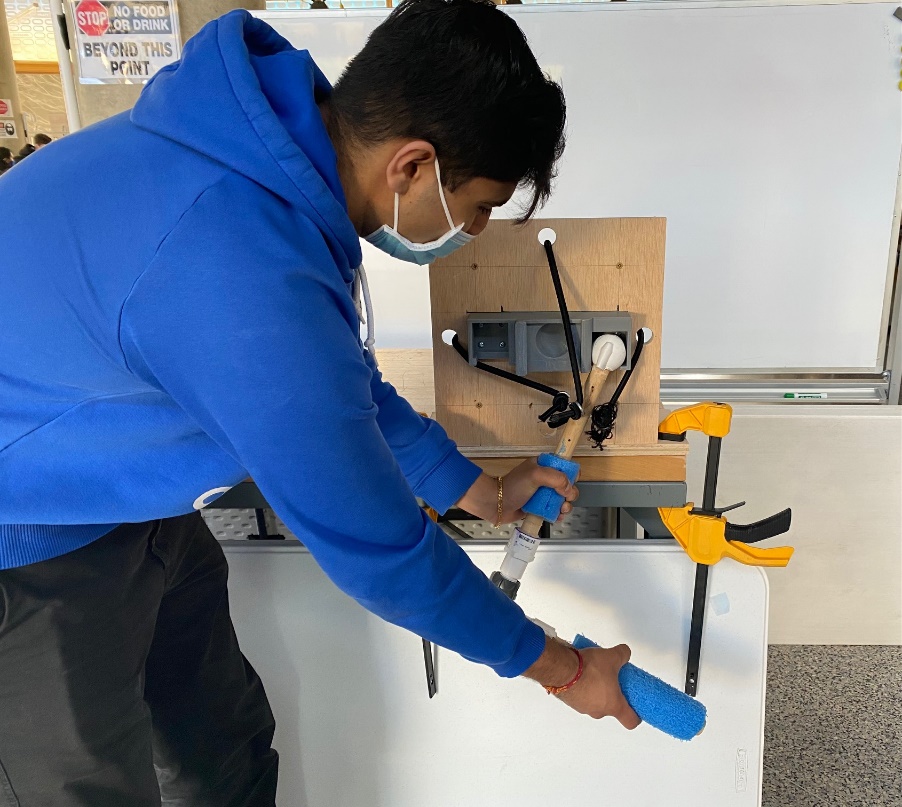


**Fig. A24**: Attempting to manipulate the humeral head to the healthy position.


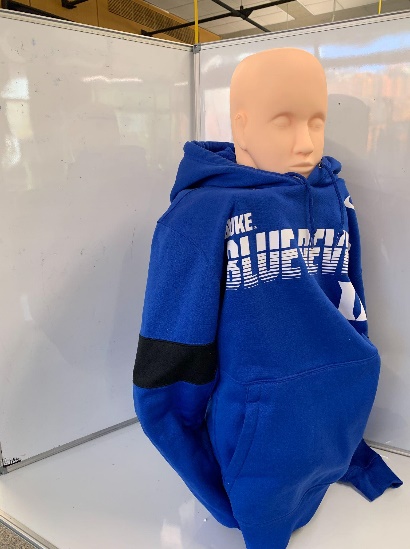


**Fig. A25**: The large sweatshirt over the model with a mannequin head on top to make the model look more realistic
